# Pharmacological Interventions for Acceleration of the Onset Time of Rocuronium: A Meta-Analysis

**DOI:** 10.1371/journal.pone.0114231

**Published:** 2014-12-02

**Authors:** Jing Dong, Lingqi Gao, Wenqing Lu, Zifeng Xu, Jijian Zheng

**Affiliations:** 1 Department of Anesthesiology, Shanghai First People's Hospital, School of Medicine, Shanghai Jiao Tong University, Shanghai, China; 2 Department of Anesthesiology, International Peace Maternal and Child Health Hospital, Shanghai, China; Scientific Inst. S. Raffaele Hosp., Italy

## Abstract

**Background:**

Rocuronium is an acceptable alternative when succinylcholine is contraindicated for facilitating the endotracheal intubation. However, the onset time of rocuronium for good intubation condition is still slower than that condition of succinylcholine. This study systematically investigated the most efficacious pharmacological interventions for accelerating the onset time of rocuronium.

**Methods:**

Medline, Embase, Cochrane Library databases, www.clinicaltrials.gov, and hand searching from the reference lists of identified papers were searched for randomized controlled trials comparing drug interventions with placebo or another drug to shorten the onset time of rocuronium. Statistical analyses were performed using RevMan5.2 and ADDIS 1.16.5 softwares. Mean differences (MDs) with their 95% confidence intervals (95% CIs) were used to analyze the effects of drug interventions on the onset time of rocuronium.

**Results:**

43 randomized controlled trials with 2,465 patients were analyzed. The average onset time of rocuronium was 102.4±24.9 s. Priming with rocuronium [Mean difference (MD) −21.0 s, 95% confidence interval (95% CI) (−27.6 to −14.3 s)], pretreatment with ephedrine [−22.3 s (−29.1 to −15.5 s)], pretreatment with magnesium sulphate [−28.2 s (−50.9 to −5.6 s)] were all effective in reducing the onset time of rocuronium. Statistical testing of indirect comparisons showed that rocuronium priming, pretreatment with ephedrine, and pretreatment with magnesium sulphate had the similar efficacy.

**Conclusion:**

Rocuronium priming, pretreatment with ephedrine, and pretreatment with magnesium sulphate were all effective in accelerating the onset time of rocuronium, and furthermore their efficacies were similar. Considering the convenience and efficacy, priming with rocuronium is recommended for accelerating the onset time of rocuronium. However, more strict clinical trials are still needed to reach a more solid conclusion due to the large heterogeneities exist among different studies.

## Introduction

Succinylcholine was once considered to be the most appropriate muscle relaxant to facilitate the endotracheal intubation during induction of anesthesia. However, it can also causes severe hyperkalemia in patients involving burns, denervation or fragile muscle membranes, severe bradycardia after a second dose of succinylcholine, increases in intracranial pressure, intragastric pressure or intraocular pressure, cardiac arrest, etc. serious side effects [1]–[4]. To avoid these fasciculation related life threatening side effects caused by succinylcholine, more and more anesthesiologists have turned to use non-depolarizing muscle relaxants to facilitate intubation[5], [6]. Many non-depolarizing muscle relaxants are currently used in clinical practice, but none of them can simultaneously satisfy the requirements of rapid onset, deep muscle relaxation, and rapid recovery for muscle relaxants to facilitate intubation as succinylcholine does [7]–[9].

With the introduction of sugammadex to rapidly reverse shallow and profound muscle relaxation induced by non-depolarizing muscle relaxants, rocuronium, a non-depolarizing muscle relaxant, has become very promising to replace succinylcholine in facilitating intubation because of its rapid onset time in non-depolarizing muscle relaxants [8], [9]. However, the onset time or the time to achieve maximum muscle block with 2ED_95_ dose of rocuronium (0.6 mg/kg) are still slower than succinylcholine (1.0 mg/kg) [9]. Increased dose of rocuronium can shorten the onset time, but it can also considerably prolong the duration of action [1], [7]. To accelerate the onset time of rocuronium-induced neuromuscular blockade, various pharmacological or non-pharmacological techniques have been used in clinical practice, but the conclusions are still inconsistent. In this paper, we mainly focused on the effects of three pharmacological intervention techniques including pretreatment with drugs, non-depolarizing muscle relaxants priming techniques, and drugs admixed with rocuronium on the onset time of rocuronium. We first searched and analyzed all the available clinical trials that studied the effects of various pharmacological interventions on the onset time of rocuronium, and then conducted meta-analysis on a select group of these pharmacological intervention techniques, and finally tried to provide anesthesiologists more reliable information in determining the most suitable protocol for safe and rapid intubation with the help of rocuronium.

## Methods

This study was carried out according to the guidelines recommended by the Cochrane Collaboration and written in accordance with the Preferred Reporting Items for Systematic Reviews and Meta-Analyses (PRISMA) statement for reporting systematic reviews [10]. We searched the Medline, Embase, Cochrane Library databases using the search terms “rocuronium” AND “onset time” to identify human studies published from year 1991 up to year 2014. According to previous literature, the time to 90,95 or 100% neuromuscular blockade defined in the eligible studies were all accepted as the onset time, and the time to the maximum blockade regardless of the percentage of neuromuscular blockade was also counted as the onset time [11]. We limited our search to clinical trials and randomized controlled trials studying the effects of pharmacological interventions on the onset time of rocuronium-induced neuromuscular blockade and published in English. The last computer search date was March 3, 2014. We collected additional relevant original papers from the reference lists of identified papers by hand search until no more relevant references were detected, and we also searched for the similar randomized controlled trials on pharmacological interventions associated with the onset time of rocuronium by searching on www.clinicaltrials.gov. Using above mentioned search strategy, we included all searched randomized controlled clinical trials that studied drug interventions to accelerate the onset time of rocuronium and were published in English with full text accessible. We excluded reviews, abstracts, protocols, and letters in our study. We used five point Oxford scale to evaluate the quality of studies [12]. As a result of multiple reporting from the same author, we compared these papers in depth and only included the most comprehensive study or the latest research in our study to minimize data overlapping from the patient population. The evaluation processes were carried out by two independent reviewers (J.D. and L.Q.G), both are anesthesiologists. Any conflicts over inclusion or exclusion were first discussed between these two reviewers, and then discussed with the senior authors until a consensus was reached. Relevant data was first extracted by J.D., and then checked by L.Q.G. The extracted relevant data included patients' characteristics, dose, timing, intervention strategy, and routes of drugs administration, onset time, which mainly came from numerical data in the text or table. If not, we extracted the relevant information from the graphs if they could be precisely estimated or contacted the original authors by email.

### Statistical analysis

Direct comparisons of intervention versus control (pairwise) and indirect comparisons between the networks of effective interventions were performed for this meta-analysis. Review Manager 5.2 software (RevMan5.2, The Cochrane Collaboration, Oxford, UK) was used for direct comparisons; ADDIS 1.16.5 (Aggregate Data Drug Information System, http://drugis.org/addis) was used for indirect comparisons. The primary outcome was the onset time of neuromuscular blockade induced by rocuronium. The effect size was the mean difference (MD). The pooled weighted MD with 95% CI was calculated using Inverse Variance statistical method based on a random effects model meta-analysis and evaluated by forest plots in RevMan 5.2. A random effects meta-analysis model assumes that the interventional effects being estimated in the different studies are not same, but follow some distributions. The statistical heterogeneity was assessed by the Cochran's Q statistic and I*^2^* value, *p*<0.10 indicated significant heterogeneity, values of I^2^<25%, ≥25%- <50%, and ≥50% were considered to represent low, modest, and large heterogeneity, respectively. However, both Q and I^2^ cannot define the source of heterogeneity, and furthermore they have only low statistical power to detect heterogeneity with small numbers of studies [13]. Therefore, we next performed subgroup analysis and prespecified combinatorial exclusion sensitivity analysis to identify the individual studies or clusters of studies which provide the strongest contribution to the heterogeneity during our meta-analysis [14]. Sensitivity analysis was applied only to those studies with an Oxford score ≥4 due to the different qualities of included trials. Potential differences were compared between the main characteristics of excluded studies and the remaining studies. Intervention techniques mainly included non-depolarizing muscle relaxants priming, drug pretreatments, drugs mixed with rocuronium, and chronic anticonvulsant therapy. Each intervention category was further divided into several subgroups depending on the characteristics of clinical trials by considering the meta-analysis results from each subgroup separately. Subgroup analyses were only performed for interventions with more than three studies. *P* values less than 0.05 (*p*<0.05) and MD without crossing the identity line were defined as statistically significant. For interventions with 10 or more studies, the effects of small studies or publication bias were visually determined by funnel plots.

For indirect comparisons, only those interventions that significantly reduced the onset time of rocuronium by direct comparison with three or more included studies were compared. Full Bayesian evidence network Meta-Analysis, using consistency or inconsistency model depending on the potential scale reduction factor (PSRF) from convergence assessment, was used to perform indirect comparison by ADDIS 1.16.5 [15]. A PSRF close to one indicates approximate convergence has been reached, and the consistency model was adopted, otherwise inconsistency model was used for further analysis [16]. The summary statistic values were presented as the mean difference with 95% confidence interval.

## Results

**Figure 1:**
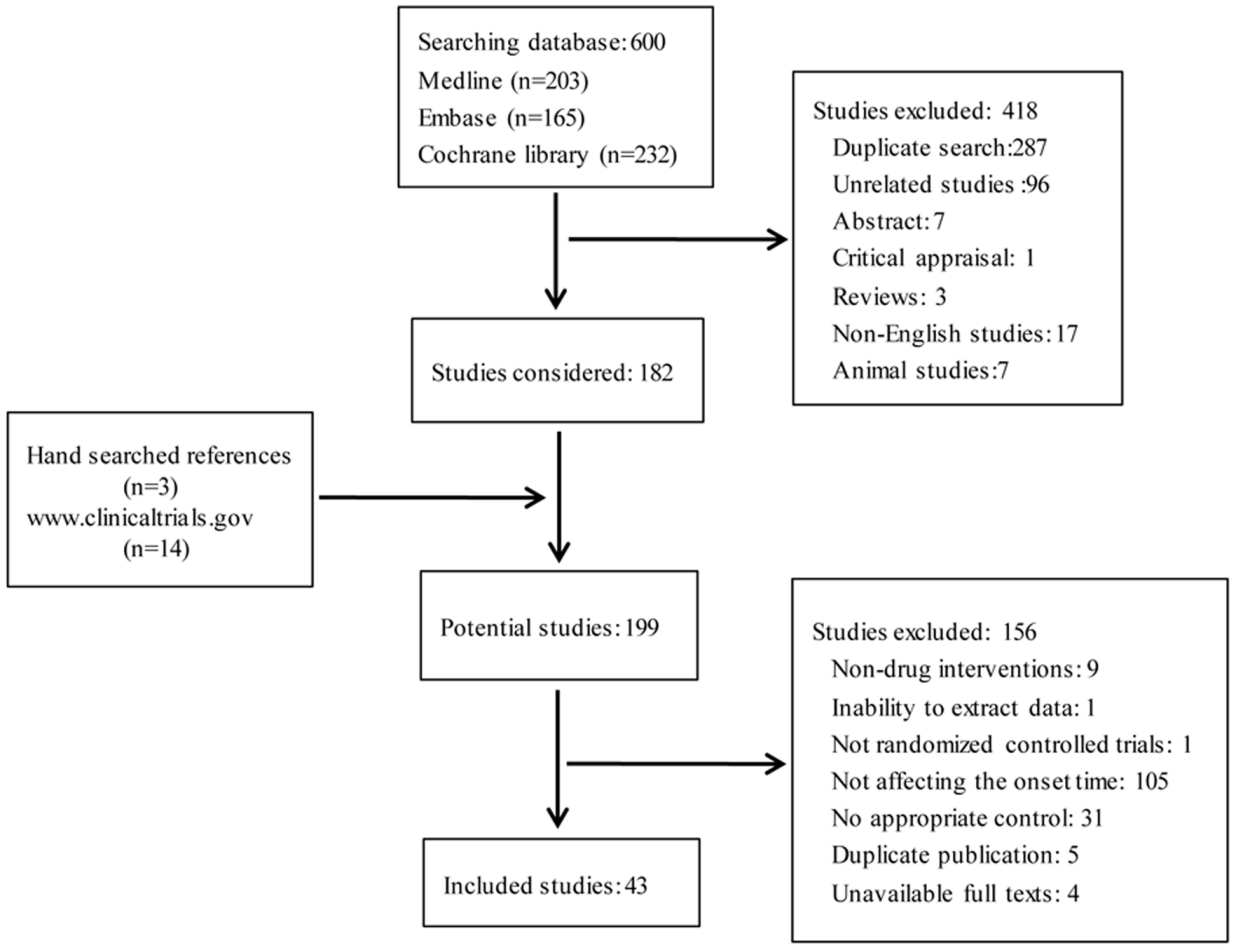
PRISMA flow diagram/

Using above mentioned search criteria, 617 potentially relevant papers were identified by searching Medline, Embase, Cochrane library databases, www.clinicaltrials.gov, and manual retrieving references of relevant papers (Fig. 1), of which 418 papers were excluded due to duplicate search (287 trials), unrelated studies (96 trials), abstracts (7 trials), critical appraisal (1 trial), reviews (3 trials), non-English studies (17 trials), animal studies (7 trials). Considering the 3 relevant papers from manual retrieval and 14 papers from searching from www.clinicaltrials.gov, 199 potentially relevant papers were further in depth analyzed, of which 156 papers were excluded, including non-drug interventions (9 trials), inability to extract data (1 trial), not randomized controlled trials (1 trial), not interventions affecting the onset time of rocuronium (105 trials), no appropriate control (31 trials), duplicate publication (5 trials), and unavailable full texts (4 trials). In the end, 43 qualified studies (2465 patients) were included in this meta-analysis. Sixteen drugs with different combinations of intervention techniques were studied to affect the onset time of rocuronium: non-depolarizing muscle relaxants priming [17]–[30]; ephedrine [31]–[35], phenylephrine [36], magnesium sulphate (MgSO4) [22], [37]–[39], suxamethonium [40], [41], lidocaine [42]–[44], midazolam [45] and esmolol [31], [35] pretreatments; rocuronium admixed with sodium bicarbonate [46], [47], mivacurium [48]–[54] and cisatracurium [55]; rocuronium priming with pretreatments of thiopentone [24], MgSO4 [22] and ketamine [29], effects of chronic anticonvulsant therapy [56]–[58] (Table 1). Intervention techniques mainly included non-depolarizing muscle relaxants priming, drug pretreatments, drugs mixed with rocuronium, and chronic anticonvulsant therapy. Each intervention category was further divided into several subcategories for detailed analysis. Subcategory analyses were only performed for interventions with more than three studies.

**Table 1:** Efficacy results of pharmacological interventions in reducing the onset time of rocuronium.

Intervention	Control	No of studies	No of patients	Mean difference* (95% CI)	Heterogeneity I^2^ (%), P value	References
**Priming with NDMRs**	No priming	13	822	−20.86 (−27.02, −14.70)	90, <0.001	20–33
Priming with rocuronium	No priming	13	792	−20.96 (−27.57, −14.34)	90, <0.001	20–33
AMG	No priming	3	121	−17.89 (−30.57, −5.20)	83, <0.001	20,26,33
MMG	No priming	4	162	−16.54 (−23.36, −9.72)	72, 0.01	22,24,28,32
EMG	No priming	3	166	−40.00 (−47.63, −32.37)	12, 0.33	25, 29,30
Priming with mivacurium	No priming	1	20	−32.00 (−38.94, −25.06)	NA	28
Priming with pancuronium	No priming	1	20	−7.6 (−17.34, 2.14)	NA	26
**Priming + ketamine**	Ketamine	1	30	1 (−20.26, 20.26)	NA	32
**Priming + thiopentone**	Thiopentone	1	30	−29.00 (−54.77, −3.23)	NA	27
**Priming + MgSO4**	No priming	1	46	−94.00 (−117.80, −70.20)	NA	25
**Pretreatment:**						
MgSO4 pretreatment	No pretreatment	4	240	−28.21 (−50.85, −5.56)	81, <0.001	25, 40–42
Ephedrine pretreatment	No pretreatment	5	282	−22.28 (−29.06, −15.50)	83, <0.001	34–38
Lidocaine pretreatment	No pretreatment	3	168	−11.90 (−27.16, 3.37)	0, 0.71	45–47
Midazolam pretreatment	No pretreatment	1	22	5.40 (-25.24, 36.04)	NA	48
Esmolol pretreatment	No pretreatment	2	62	25.21 (20.10, 30.33)	0, 0.83	34,38
Suxamethonium pretreatment	Sham	2	54	-18.09 (-20.20, -15.97)	35, 0.21	43,44
Phenylephrine pretreatment	Saline	1	64	12.00 (4.10, 19.90)	NA	39
**Admixture**						
Mivacurium admixture	No admixture	7	459	-1.46 (-16.64, 13.72)	93, <0.001	51–57
<0.07 mg/kg	No admixture	3	86	9.55 (−2.31, 21.41)	45, 0.14	51, 52, 54
0.07–0.15 mg/kg	No admixture	7	373	−1.21 (−20.03, 17.60)	94, <0.001	51–57
Sodium bicarbonate admixture	No admixture	2	120	−40.12 (−54.32, −25.92)	89, 0.002	49, 50
Cisatracurium admixture	No admixture	1	38	6.00 (−24.52, 36.52)	NA	58
**Anticonvulsant therapy**	No therapy	3	134	24.02 (1.67, 46.37)	0, 0.84	59–61

NA: Not applicable; NDMRs: non-depolarizing muscle relaxants; AMG: acceleromyography; MMG: mechanomyography; EMG: electromyography; MgSO4: magnesium sulphate; *Random effects model

**Figure 2:**
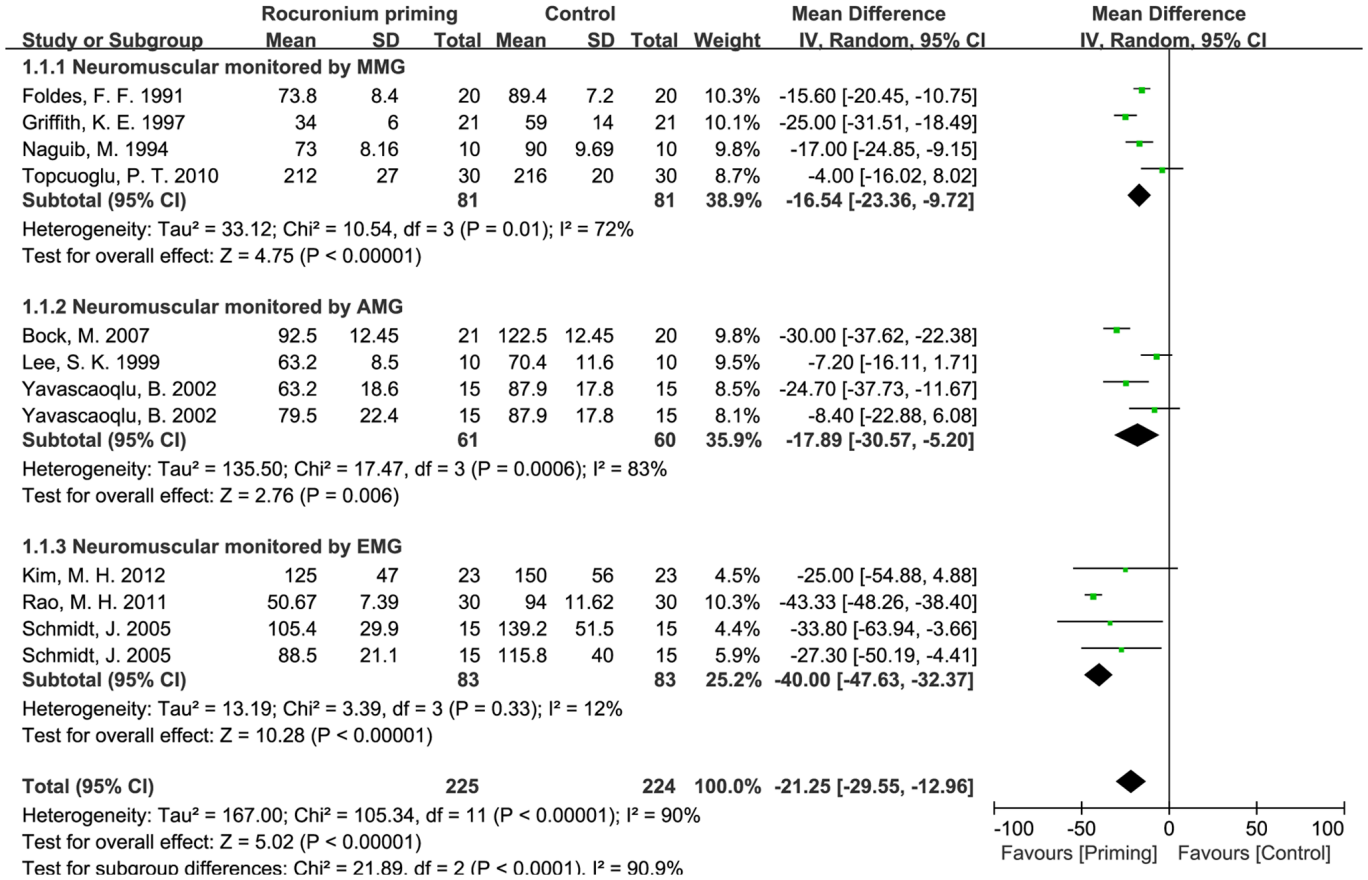
Effects of rocuronium priming on the onset time of rocuronium. Administration protocol of rocuronium priming: 0.06 mg/kg rocuronium was first intravenously administrated as priming dose, then 0.54 mg/kg rocuronium i.v.

**Figure 3:**
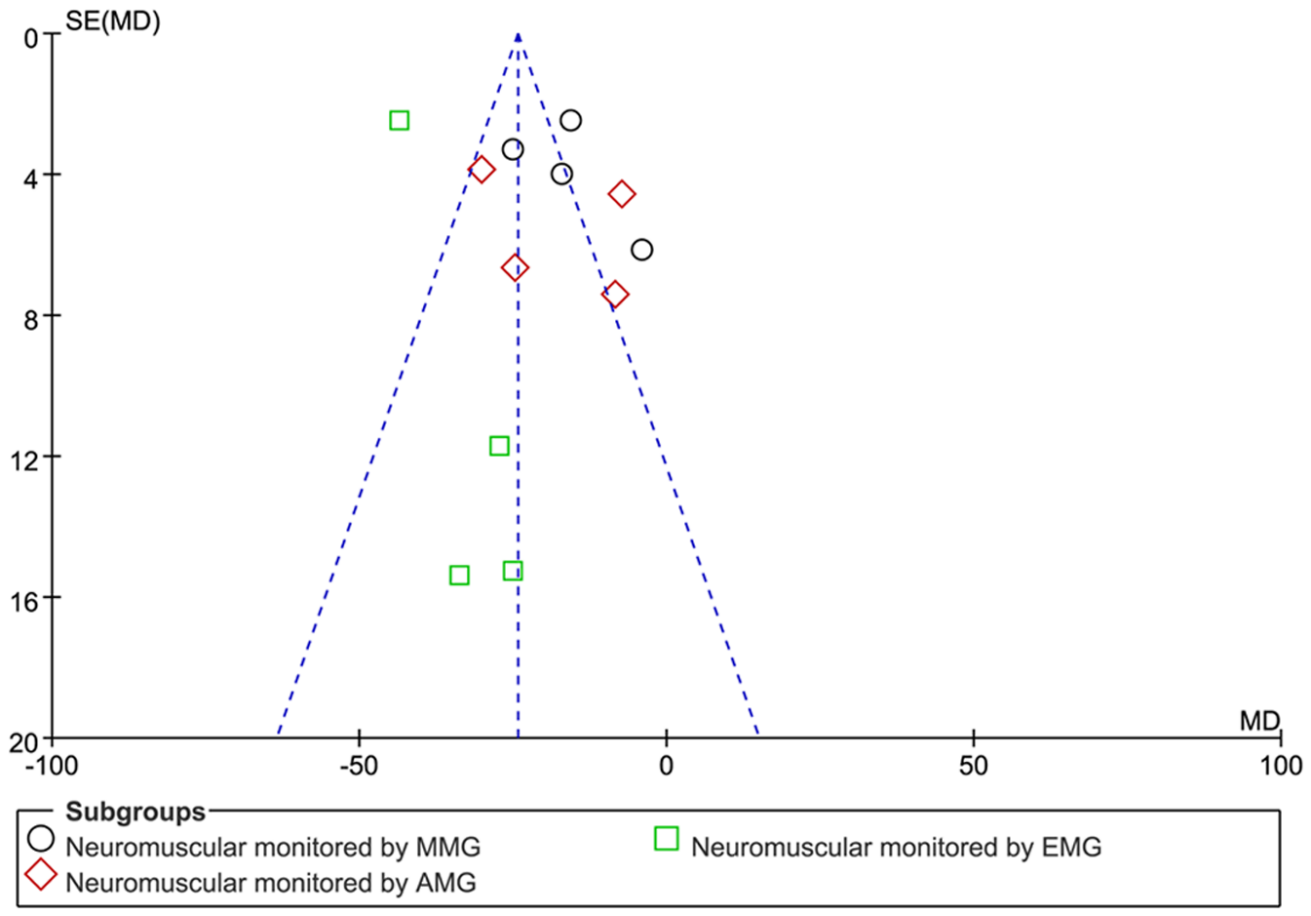
Funnel plot of studies based on rocuronium priming. AMG: acceleromyography; MMG: mechanomyography; EMG: electromyography; Administration protocol of rocuronium priming: 0.06 mg/kg rocuronium was first intravenously administrated as priming dose, then 0.54 mg/kg rocuronium i.v.

Non-depolarizing muscle relaxant priming is the most commonly used intervention to study the onset time of rocuronium, and rocuronium itself is the most commonly used priming agent with 13 studies, other studied non-depolarizing muscle relaxant priming agents include mivacurium (1 trial) and pancuronium (1 trial). The pooled mean difference of non-depolarizing muscle relaxant priming in the onset time of rocuronium was −20.9 s (95% CI: −27.0 to −14.7 s, *p*<0.01), indicating that non-depolarizing muscle relaxants priming can shorten the onset time of rocuronium. However, the I^2^ value of heterogeneous test was much higher (about 90%) (Table.1). In order to reduce the possible bias of our conclusion caused by large heterogeneity, subgroup analyses were performed. We focused on the effects of rocuronium priming on the onset time of rocuronium in 13 RCTs with 792 patients since there was only one study for mivacuronium priming and one for pancuronium priming. 11 out of 13 RCTs with rocuronium priming used the protocol of 0.06 mg/kg rocuronium priming followed by 0.54 mg/kg rocuronium injection. We next performed subgroup analysis on these 11 RCTs according to the different methods of neuromuscular monitoring [electronmyography (EMG), mechanomyography (MMG) and acceromyograph (AMG)]. The mean differences with rocuronium priming were −40.0 s (−47.6 to −32.4 s, *p*<0.01) measured by EMG, −17.9 s (−30.6 to −5.2 s, *p*<0.01) by AMG, and −16.5 s (−23.4 to −9.7 s, *p*<0.01) by MMG, and the mean difference of overall effect of rocuronium priming was −21.0 s (−27.6 to −14.3 s, *p*<0.01) (Fig.2). Statistical heterogeneity I^2^ in MMG, AMG and EMG subgroups were about 72% (*p* = 0.01), 83% and (*p*<0.01) and 12% (*p* = 0.03), indicating the heterogeneity of different datasets in MMG and AMG subgroups. The funnel plot of subgroups demonstrated asymmetry, suggesting the existence of small studies effect or publication bias for this intervention (Fig. 3), so fixed and random-models were compared, and the result of meta-analysis did not show any difference. Sensitivity analysis indicated that the meta-analysis results were not changed if only studies with Oxford score ≥4 were included.

**Figure 4:**
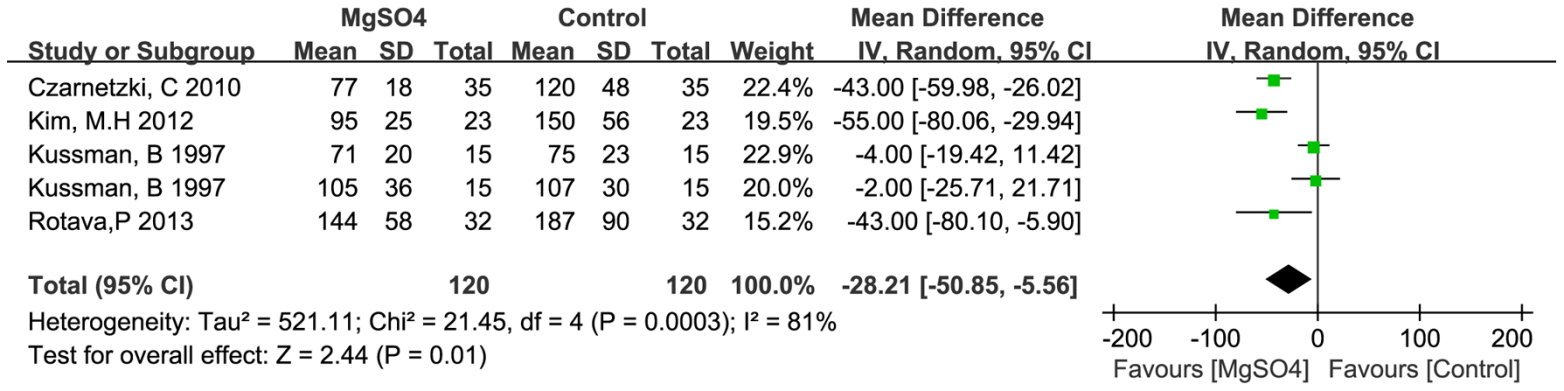
Effects of pretreatment with magnesium sulphate on rocuronium onset time.

**Figure 5:**
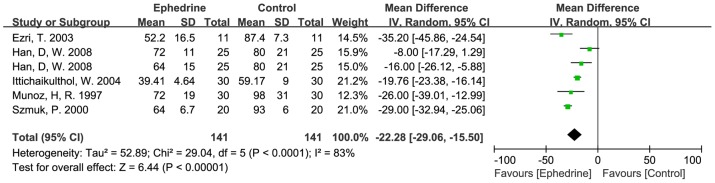
Effects of pretreatment with ephedrine on rocuronium onset time.

**Figure 6:**
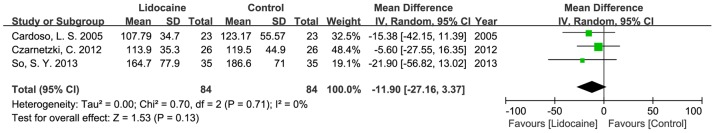
Effects of pretreatment with lidocaine on rocuronium onset time.

Pretreatments with MgSO4 and ephedrine were also significantly effective in accelerating the onset time of rocuronium. The mean difference for MgSO4 pretreatment and ephedrine pretreatment were −28.2 s (−50.9 to −5.6 s, *p*<0.05), −22.3 s (−29.1 to −15.5 s, *p*<0.01) individually (Fig.4, Fig.5). Pretreatment with lidocaine showed no significant effect in accelerating the onset time of rocuronium in three trials, the mean difference was −11.9 s (−27.2 to 3.4 s, *p*>0.05) (Fig. 6). However, studies were heterogeneous, as indicated byI^2^ values of heterogeneous tests in pretreatment with MgSO4 and pretreatment with ephedrine were significant high (about 81% and 83%) (Fig.4, Fig.5). The sources of heterogeneity might originate from different doses, timing, different monitoring methods of neuromuscular block among these studies, etc., but there are not enough studies to do subgroups so far.

**Figure 7:**
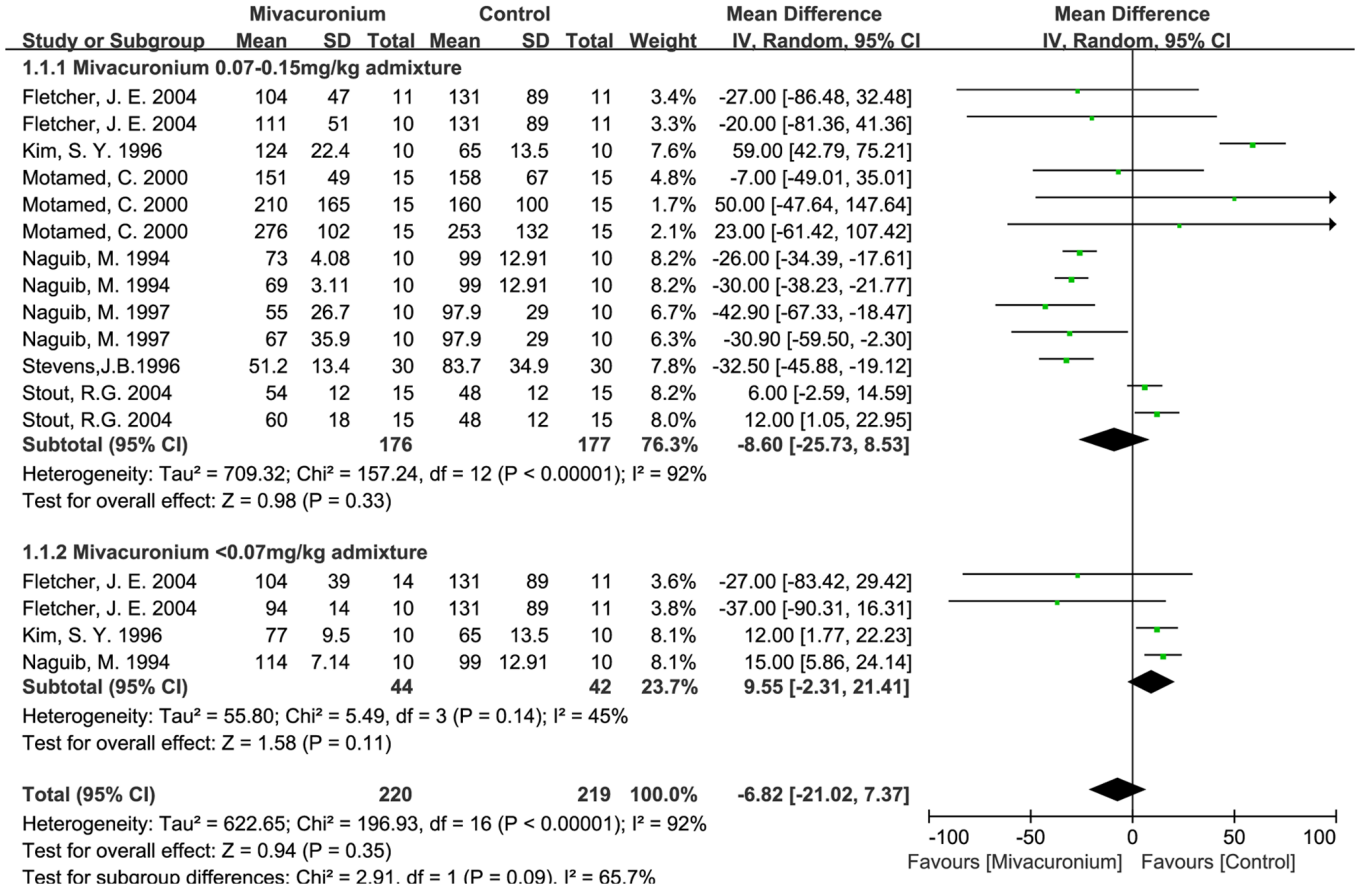
Effects of admixture with mivacurium on rocuronium onset time.

Rocuronium admixed with mivacurium also showed no significant effect in reducing the onset time of rocuronium, the mean difference was −1.5 s (−16.6 to 13.7 s, *p*>0.05). Considering the different admixture dosages of mivacurium, subgroups analyses (0.07–0.15 mg/kg and ≤0.07 mg/kg mivacurium groups) were further analyzed, but no significant differences were found compared to all effect sizes (Fig. 7). However, sensitivity analysis showed that two studies (Kim, 1996 and Stout, 2004) [49], [54]have a significant effect on the heterogeneity and effect size. When these two studies were excluded, the I^2^ value from the subgroup of rocuronium admixed with mivacurium (0.07–0.15 mg/kg) (five trials left) became zero percent instead of 94%, and rocuronium admixed with mivacurium (0.07–0.15 mg/kg) became effective (*p*<0.05) in reducing the onset time of rocuronium, indicating that these two trials might be the major sources of heterogeneity.

To rank the efficacy of interventions, a network meta-analysis was performed on three statistically significant pairwise comparisons using ADDIS software: priming with rocuronium versus control; pretreatment with MgSO4 versus control; pretreatment with ephedrine versus control (Table. 2). Indirect comparisons were derived from 4 chains in 34 studies. The convergence plot of interventions versus control showed that the PSRFs were quite far away from one in the beginning, close to one after 5,000 iterations, and the convergence was very stable after 50,000 iterations, which indicated that the convergence has been reached and consistency model could be adopted for further network meta-analysis [16]. Table.2 included the mean differences with 95% confidence intervals for all treatments relative to each other under the consistency model. The mean differences with 95% confidence intervals were: priming with rocuronium versus pretreatment with ephedrine was −1.5 s (−14.2 to 11.4 s, *p*>0.05), pretreatment with ephedrine versus pretreatment with MgSO4 was 4.6 s (−14.2 to 24.0 s, *p*>0.05), and priming with rocuronium versus pretreatment with MgSO4 was −6.1 s (−22.5 to 10.4 s, *p*>0.05). So priming with rocuronium, pretreatment with MgSO4 and pretreatment with ephedrine had similar efficacy in reducing the onset time of rocuronium. Direct comparisons results were also retrieved in this network meta-analysis model. Since the direct comparison only involved two trials, resulting in a much larger confidence interval [59]. As the methods of estimation are different, the direct comparisons of mean difference values from RevMan and ADDIS differ slightly.

**Table 2:** Indirect comparisons between effective pharmacologic interventions.

	Control	Rocuronium priming	MgSO4 pretreatment	Ephedrine pretreatment
Control	1.00			
Rocuronium priming	−20.85(−14.57, 7.01)*	1.00		
MgSO4 pretreatment	−26.85(−11.37, −42.19) *	−6.05(−22.86, 10.60)	1.00	
Ephedrine pretreatment	−22.25(−11.10, −33.80)*	−1.46 (−14.23, 11.35)	−4.63(14.31, −23.75)	1.00

This net-work analysis was performed by ADDIS software using Markov chain Monte Carlo (MCMC) method rather than estimation in Review Manager. Mean difference of each intervention against control was slightly different from that of direct comparison in Table 1. MgSO4: Magnesium sulphate; *p<0.05.

## Discussion

Sixteen drugs with different combinations of intervention techniques were identified in this meta-analysis for the onset time of rocuronium, but only rocuronium priming, pretreatment with MgSO4 and pretreatment with ephedrine were effective in shortening the onset time of rocuronium. Pretreatment with lidocaine and admixture with mivacurium were needed to be further analyzed. Most of other interventions had only one to two randomized clinical trials, therefore, more randomized clinical trials are needed before in depth meta-analysis.

Due to the characteristics of rapid onset and complete reversal of profound neuromuscular blockade by sugammadex, rocuronium has become a useful alternative to succinylcholine for facilitating the endotracheal intubation during rapid sequence induction in certain situations [8], [60]. However, the onset time of rocuronium used for producing good intubation condition is still slower than that of succinylcholine, the average onset time of rocuronium at 2×ED_95_ dose without drug interventions is about 102.4±24.9 s. This meta-analysis demonstrated that priming with rocuronium, pretreatment with MgSO4 or ephedrine are all effective in terms of reducing the onset time of rocuronium, and furthermore, they have similar efficacy. Since only three related studied were identified in this analysis, more studies about the effect of lidocaine pretreatment on the onset time of rocuronium are needed to confirm our result. Rocuronium priming can effectively reduce the onset time of rocuronium (MD: −21.0 s, 95% CI: −27.6 to −14.3 s), but rocuronium admixed with mivacurium (a non-depolarizing muscle relaxant) did not affect the onset time of rocuronium (MD: −1.5 s, 95% CI: −16.6 to13.7 s). Further sensitivity analysis showed that rocuronium admixed with mivacurium (0.07–0.15 mg/kg) in five studies was effective (*p*<0.05) in reducing the onset time of rocuronium after the two trials Kim, 1996 and Stout, 2004 were excluded, suggesting more studies about the effects rocuronium admixed with mivacurium on rocuronium onset time are still needed to elucidate this issue. While considering convenience and efficacy, priming with rocuronium is a recommended for accelerating rocuronium onset of action during anesthesia induction.

This study also has some limitations. We excluded non-English articles in this study. Although language-restricted meta-analyses have been repeatedly reported to cause no bias for estimating the effectiveness of different interventions [61], one should still be cautious in considering our conclusions to be final especially when some interventions only involved a few high qualified studies published in English. The definition of onset time was derived from the previous report [11], but the range was broad,the time to 90, 95 or 100% neuromuscular blockade defined in the eligible studies were all accepted as the onset time, and the time to the maximum blockade regardless of the percentage of neuromuscular blockade was also counted as the onset time, which might lead to large heterogeneity in our study. In addition, the models or devices of neuromuscular stimulation and detection were different, which might also cause large heterogeneity. Some interventions including sodium bicarbonate, ketamine and suxamethonium reached statistical significance, but they cannot readily be performed with meta-analysis because of lacking enough clinical trials. Further studies are needed to elucidate the efficacy of these interventions and their underlying mechanisms.

In conclusion, this meta-analysis suggests that priming with rocuronium, pretreatment with MgSO4 and pretreatment with ephedrine can effectively reduce the onset time of NMB induced by rocuronium. Considering the efficacy and convenience, priming with rocuronium is recommended for accelerating rocuronium onset of action during anesthesia induction. Due to the relatively large heterogeneity and small effect size in some subgroups, more randomized controlled clinical trials are still needed to get a solid recommendation for clinical use no matter for a most appropriate approach for monitoring of blockade and definition of onset time or doses of interventional drugs and methods or different populations and different pathophysiological conditions.

## Supporting Information

Checklist S1 **PRISMA Checklist.**
(DOC)
